# The Impact of Urinalysis Turnaround Times on Patient Disposition in the Emergency Department

**DOI:** 10.5811/westjem.53952

**Published:** 2026-07-16

**Authors:** DaMarcus E. Baymon, Patricia Hernández, Paul Chen, Leon D. Sanchez, Dana Im, Alice Bukhman, Joshua W. Joseph

**Affiliations:** * Harvard Medical School, Brigham and Women’s Hospital, Department of Emergency Medicine, Boston, Massachusetts; † Harvard Medical School, Massachusetts General Hospital, Department of Emergency Medicine, Boston, Massachusetts

## Abstract

**Introduction:**

Diagnostic efficiency in the emergency department (ED) is crucial for workflow. Among these, urinalysis (UA) is a key test for abdominopelvic complaints. Prolonged UA turnaround times can extend patient length of stay and reduce patient satisfaction, highlighting the need for improvement. In this study qw sought to measure the correlation between UA turnaround times and time to disposition.

**Methods:**

We conducted a retrospective analysis of discharged ED patients with abdominopelvic complaints who had a UA ordered between January–August 2022. Electronic health records data included UA order-to-result time, urine human chorionic gonadotropin (hCG), time to disposition, and complete blood count (CBC) turnaround time as a venipuncture comparator. Spearman correlation coefficients assessed associations between lab processing times and time to disposition.

**Results:**

Among 6,708 discharged patients included in the study, 3,795 presented with abdominopelvic complaints. In this subgroup, 3,688 UAs, 1,285 urine hCG tests, and 2,966 CBCs were obtained. Median times from UA order-to-specimen receipt and result were 65 (IQR 29–138) and 92 (53–168) minutes, respectively. A modest positive correlation was observed between UA order-to-result time and time to disposition (ρ = 0.46), relative to CBC order-to-result time (ρ = 0.29). The relationship was slightly weaker for urine hCG (ρ = 0.35). Laboratory turnaround times showed minimal correlation with ED time to disposition (UA ρ = 0.04; urine hCG ρ = 0.03; CBC ρ = 0.09).

**Conclusion:**

Urinalysis acquisition and processing times are modestly correlated with time to disposition in patients with abdominopelvic complaints. Reducing collection time for UA and urine hCG tests may improve throughput for this patient cohort.

## INTRODUCTION

Abdominal pain, dysuria, hematuria, and flank pain are among the most common emergency department (ED) presentations. Genitourinary diagnoses account for 8 million of 139 million ED visits per year suggesting that rapidly obtaining urinalyses (UA) may support efficient ED operations.^1^,^2^ Delays in UA turnaround times may prolong disposition for patients who are otherwise ready for discharge. Longer ED length of stay is associated with lower satisfaction, worse outcomes, and increased costs.^3^–^5^

Relative to expensive, institution-level measures, such as increasing inpatient bed availability and staffing, reducing laboratory turnaround times is a feasible way to address delays.^6^ However, obtaining urine tests poses logistical challenges because patients must have urine available and must participate in collection or catheterization. Prior studies suggest that faster turnaround times improve patient flow and reduce ED congestion, yet the specific impact of UA turnaround times on disposition remains unclear.^7^ A recent study found no improvement in ED time to disposition after implementing a urine collection triage system, although the sample size was limited.^8^ Thus, the objective of our study was to measure the correlation between UA collection and turnaround times and time to disposition among ED patients with abdominopelvic complaints.

## METHODS

We conducted this retrospective study from January–August 2022 in a single urban ED with an annual volume of approximately 32,000 visits. The institutional review board exempted the study from informed consent since only anonymous data were used. Eligible participants were adults (≥18 years of age) discharged with abdominopelvic complaints (including “abdominal pain,” “flank pain,” “hematuria,” “dysuria,” “pelvic pain,” “vaginal bleeding,” and “vaginal discharge”) who had an order for UA or urine human chorionic gonadotropin (hCG). Electronic health records (EHR) data included timestamps of lab test orders, specimen receipts, test result times, and time to disposition. These totals reflect tests performed rather than unique patients, as some patients underwent multiple tests. Orders were all clinician-initiated because no triage protocol-based urine ordering was in place during the study period.

We defined order time as the test order entry timestamp, specimen receipt time as the laboratory accession timestamp, and result time as the final result reporting timestamp in the EHR. Lab turnaround time was defined as the interval between specimen receipt and result reporting. Time to disposition was defined as the interval between ED arrival and disposition decision timestamp. Disposition decision time was abstracted from EHR timestamps corresponding to the time at which the treating clinician entered the patient’s disposition. We abstracted complete blood count (CBC) collection times as a proxy for venipuncture-based tests.

We followed recommended methods for medical record reviews including explicit inclusion and exclusion criteria, predefined variable definitions, and a standardized data abstraction process as outlined by Worster and Bledsoe.^9^ The primary outcome was the correlation between UA turnaround time and disposition time. Secondary outcomes included the correlations between UA order-to-receipt interval, urine hCG processing times, and CBC turnaround time with disposition time.

Descriptive statistics, including medians with IQR, were computed for lab collection times and disposition times. We assessed normality with the Shapiro-Wilk test. Lab testing and collection intervals were compared using the Kruskal-Wallis test across the three test groups followed by Mann-Whitney-U testing between groups of pairs when significant. Spearman correlation coefficients were used to assess associations between lab collection times and disposition times. A two-sided *P* value of .01 was considered significant.

**Population Health Research Capsule**

What do we already know about this issue?

*Urinalysis (UA) testing may prolong emergency department (ED) times to disposition. However, the relative impact of urine collection lab processing is unclear*.

What was the research question?

*Is ED time to disposition correlated with urinalysis processing intervals?*

What was the major finding of the study?

*UA order-to-result time was correlated with disposition time (ρ = 0.46); each 30-minute delay added seven minutes to disposition time*.

How does this improve population health?

*Targeting urine collection delays may reduce time to disposition*.

## RESULTS

A total of 6,708 patients were discharged during the study period. Of those, 3,795 patients (2,620 female, 69%) presented to the ED with abdominopelvic complaints between January–August 2022. A total of 3,688 UAs, 1,285 urine hCG tests, and 2,966 CBCs were obtained. These counts reflect tests performed rather than unique patients because some encounters included multiple tests; however, all three tests reflect independent orders, specimens, and reporting personnel at our institution. None of the laboratory intervals demonstrated normal distributions. Significant differences were observed between UA, urine hCG, and CBC for order-to-specimen receipt (Kruskal-Wallis H = 132, *P* < .001), order-to-result (H = 173, *P* < .001), and laboratory turnaround time (H = 427, *P* < .001). Urinalyses consistently took longer than any other test. The median time from the order being placed to specimen receipt for UA was 65 minutes (interquartile range [IQR] 29–138), while the median time from order-to-result was 92 minutes (53–168), encompassing a median lab turnaround time of 20 minutes (13–31), all of which were significant relative to urine hCG and CBC (*P* < .01 for all comparisons).

**Figure 1:**
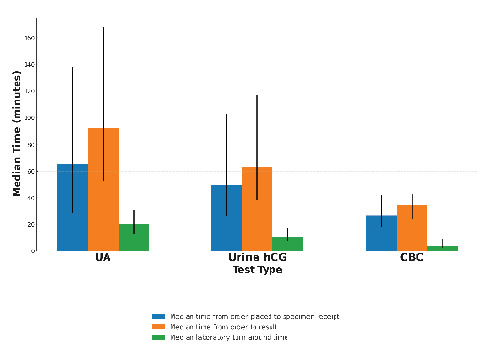
Median time (in minutes) for urinalysis, urine human chorionic gonadotropin, and complete blood count in a retrospective study of emergency department patients with abdominopelvic complaints that examined the association between laboratory turnaround times and time to patient disposition. Error bars represent interquartile ranges.*CBC*, complete blood count; *UA*, urinalysis.

Urine hCG orders consistently took longer to complete than CBCs, with a median time from order-to-specimen receipt of 49 minutes (26–103), median time from order to result of 63 minutes (IQR 38–117), and median lab turnaround time of 11 minutes (8–17) (*P* < .01 for all comparisons). The median times for CBCs were relatively rapid with median time of order-to-specimen receipt of 27 minutes (18–42), median time from order. to result of 35 minutes (24–43), and median turnaround time of 4 minutes (3–9). Time from clinician sign-up to order placement was rapid for Uas: median 5 minutes (3–6) and did not differ significantly compared to other tests. Median laboratory processing times for UA, urine hCG, and CBC are summarized in Figure 1.

Spearman correlation analysis demonstrated a positive relationship between UA order to result and time to disposition (ρ = 0.46). The correlation between urine hCG test ordering and result times and time to disposition was more modest (ρ = 0.9), while the CBC showed a weaker correlation (ρ = 0.29). All correlations were statistically significant. Lab turnaround times showed little to no correlation with time to disposition TTD for UA (ρ = 0.04), urine hCG (ρ = 0.03), and CBC (ρ = 0.09), and were not statistically significant, nor were intervals from clinician sign-up to order placement. A simple regression analysis of UA order-to-result times demonstrated that every additional 30-minute delay beyond the median order-to-result time was associated with an additional seven-minute delay in TTD.

## DISCUSSION

This retrospective study demonstrated a modest, positive correlation between UA collection times and time to disposition in the ED, with a weaker trend for urine hCG tests. These findings align with prior work by Neyman et al. who reported that UA orders extended the ED length of stay by 35–45 minutes, and that time to disposition nearly doubled when both UAs and blood test were ordered, suggesting an additive effect.^10^ Anad et al similarly found that UA orders were associated with increased time to disposition among discharged patients.^11^ Together, these findings highlight the role of testing efficiency in patient throughput and ED operations.

Prior work evaluating lab turnaround time more broadly has demonstrated that improvements in turnaround time are associated with clinically meaningful reductions in ED length of stay. In a large retrospective analysis, Kaushik et al. reported that a 1-minute decrease in lab turnaround time was associated with a 0.50-minute decrease in ED length of stay, and Vrijsen et al similarly found that a one-minute increase in laboratory TAT was associated with a 0.32-minute increase in ED length of stay after adjustment for covariates.^12^,^13^ Our findings, focused on urine tests, suggests that delays relevant to time to disposition may occur predominantly before lab processing. However, given the observational design of our study and the absence of key operational covariates, delays in UA turnaround time should be interpreted as an associated marker of encounter complexity and workflow, rather than a direct casual driver of prolonged time to disposition.

While UAs required more time relative to other tests in this study, most delays occurred during specimen acquisition. Reducing collection time may improve throughput and patient satisfaction.^14^, ^15^ Although UA point-of-care testing could shorten order-to-result time, these results suggest that operational improvements in urine specimen collection, such as providing restroom instructions or standardizing collection when rooming patients, may also be effective. Enhancing communication among clinicians, nursing staff, emergency service assistants, and lab personnel may streamline workflow and reduce delays. Future studies should examine the impact of targeted UA ordering algorithms and automated notification systems on workflow and time to disposition.

While some studies indicate a clear impact, others suggest that factors beyond UA collection may play a greater role in ED throughput. Despite their frequent use, UAs do not consistently impact clinical decision-making, with up to 33% of results not influencing medical decisions, 25% not affecting disposition decisions, and 17% of UAs being canceled.^11^ A 2020 randomized controlled trial found no difference in time to disposition between patients who provided urine samples after physician evaluation and those who provided samples in triage.^8^ This inconsistency highlights the need to reconsider routine UA use in the ED to optimize time to disposition metrics and alleviate unnecessary burdens on patients and ED staff.

## LIMITATIONS

This study has several limitations. Its retrospective nature limits causal inference. This preliminary study did not adjust for factors that may delay urine acquisition independent of lab performance, such as inability to provide a specimen due to pain, dehydration, or frailty, parallel diagnostic testing, or ED crowding and staffing, which may influence both collection and disposition timing. Future studies should measure and adjust for these clinical and operational drivers to better isolate the association between UA timing intervals and time to disposition. Additionally, conducting the study at a single institution and focusing on discharged patients reduces generalizability and may select for a less acute population with shorter time to disposition than admitted patients.

Due to the de-identification process, our dataset did not include patient age as a covariate; thus, it is likely that for older patients with significant frailty, the logistics of obtaining a clean-catch UA relative to a catheterization could contribute significantly to the time needed to obtain a specimen. Similarly, while CBCs served as a proxy for venipuncture-based testing, chemistry panels may represent a more consequential prerequisite for imaging.

## CONCLUSION

Delays in obtaining urinanalyses demonstrated a modest association with delays in patient throughput, and UAs took considerably longer than urine and blood tests ordered contemporaneously. Conversely, while UAs took longer to analyze, no lab turnaround times were associated with throughput for any test. These findings indicate that expediting UA collection may have an outsized effect on ED throughput relative to improving other tests. They also suggest a salient target for intervention, as meaningful improvements may result from improving collection practices and communication, rather than from faster lab equipment or increased staffing. Given the frequency with which urine studies are obtained in the ED, even modest reductions in urine acquisition delays may translate into significant improvements in ED throughput and patient experience. Further multicenter and prospective studies are needed to generalize these findings and evaluate future interventions.
